# Uncertainty on the effectiveness and safety of rivaroxaban in premenopausal women with atrial fibrillation: empirical evidence needed

**DOI:** 10.1186/s12872-017-0692-1

**Published:** 2017-10-13

**Authors:** Herbert J. A. Rolden, Angela H. E. M. Maas, Gert Jan van der Wilt, Janneke P. C. Grutters

**Affiliations:** 1 Council for Public Health and Society, The Hague, The Netherlands; 2 Department for Health Evidence, Radboud University Medical Center, Nijmegen, The Netherlands; 3 Department of Cardiology, Radboud University Medical Center, Nijmegen, The Netherlands

**Keywords:** Abnormal uterine bleeding, Atrial fibrillation, Premenopausal women, Rivaroxaban, Value of information, Vitamin K antagonists

## Abstract

**Background:**

Novel anticoagulations (NOACs) are increasingly prescribed for the prevention of stroke in premenopausal women with atrial fibrillation. Small studies suggest NOACs are associated with a higher risk of abnormal uterine bleeds than vitamin K antagonists (VKAs). Because there is no direct empirical evidence on the benefit/risk profile of rivaroxaban compared to VKAs in this subgroup, we synthesize available indirect evidence, estimate decision uncertainty on the treatments, and assess whether further research in premenopausal women is warranted.

**Methods:**

A Markov model with annual cycles and a lifetime horizon was developed comparing rivaroxaban (the most frequently prescribed NOAC in this population) and VKAs. Clinical event rates, associated quality adjusted life years, and health care costs were obtained from different sources and adjusted for gender, age, and history of stroke. A Monte Carlo simulation with 10,000 iterations was then performed for a hypothetical cohort of premenopausal women, estimated to be reflective of the population of premenopausal women with AF in The Netherlands.

**Results:**

In the simulation, rivaroxaban is the better treatment option for the prevention of ischemic strokes in premenopausal women in 61% of the iterations. Similarly, this is 98% for intracranial hemorrhages, 24% for major abnormal uterine bleeds, 1% for minor abnormal uterine bleeds, 9% for other major extracranial hemorrhages, and 23% for other minor extracranial hemorrhages. There is a 78% chance that rivaroxaban offers the most quality-adjusted life years. The expected value of perfect information in The Netherlands equals 122 quality-adjusted life years and 22 million Euros.

**Conclusions:**

There is a 22% risk that rivaroxaban offers a worse rather than a better benefit/risk profile than vitamin K antagonists in premenopausal women. Although rivaroxaban is preferred over VKAs in this population, further research is warranted, and should preferably take the shape of an internationally coordinated registry study including other NOACs.

**Electronic supplementary material:**

The online version of this article (10.1186/s12872-017-0692-1) contains supplementary material, which is available to authorized users.

## Background

Atrial fibrillation (AF) is the most common cardiac arrhythmia and is a chronic or recurrent illness that greatly affects patients’ quality of life. It is estimated that over 2% of people suffer from AF and its prevalence is expected to increase, in part due to population ageing in combination with a deterioration of lifestyle factors such as overweight, leading to more diabetes, hypertension and ischemic heart disease at a young age [1–3]. AF drastically increases the risk of ischemic stroke, and Vitamin K Antagonists (VKAs) have been prescribed for decades to prevent such stroke events in patients with AF. Unfortunately, VKAs are associated with serious side-effects, of which intracranial hemorrhage is the most severe, causing extremely high rates of emergency hospital admissions [4], requiring regular monitoring for dose titration [5].

Since 2010, four different pharmaceutical agents have entered the market as an alternative to VKAs: dabigatran, rivaroxaban, apixaban and edoxaban. Their phase III trials suggest that these novel oral anticoagulants (NOACs) are at least non-inferior to VKAs in terms of effectiveness, and are associated with a lower risk of intracranial hemorrhages [6–10]. Another important benefit of NOACs is that they are provided in a standard dose and do not require frequent monitoring. Results from observational studies suggest that rivaroxaban (Rvx) is the most prescribed NOAC, at least in Canada and the UK [11, 12]. A plausible reason for its popularity is that RVX is taken once daily, where other NOACs have a twice daily dose regimen [13].

Regardless of the advantages that RVX provides, some researchers and clinicians still have reservations in clinical practice. A main problem with RVX is that - as for other NOACs - there is a lack of empirical evidence on its benefit/risk profile in certain patient subgroups, and premenopausal women form a marked example, for whom an important neglected factor is that RVX is associated with a higher risk of abnormal uterine bleeds (AUBs) than VKAs [14, 15]. It is therefore possible that RVX might be the “wrong” treatment choice for premenopausal women, and that its widespread use in this subgroup may cause more harm than benefit, especially considering that heavy and irregular menstrual bleeding is common in women in their forties and requires specific attention [16].

As premenopausal women have not been separately investigated, we aim to assess the impact of RVX prescription in this subgroup by synthesizing and modeling all relevant indirect empirical evidence that is currently available. We have done this by simulating clinical event rates from the phase III trial on RVX, and the different subgroup analyses performed on this trial (sometimes adjusted using additional empirical evidence), and the consequences of these events in a hypothetical cohort of premenopausal women in a specific model. An adjoining value-of-information analysis shows whether further research in this subgroup is warranted.

## Methods

### Model description

**Fig. 1:**
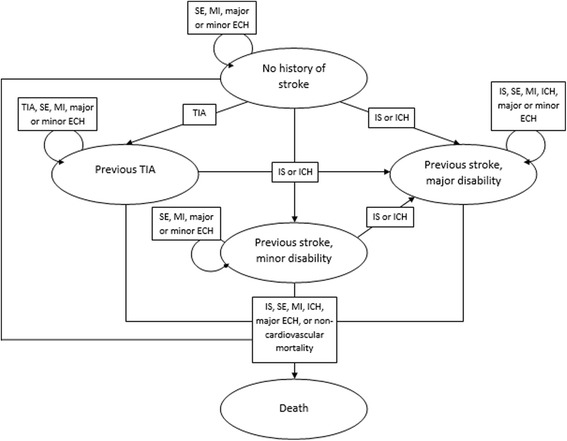
Illustration of the Markov model with health states (circles), clinical events (rectangles), and transition possibilities (arrows). Abbreviations: *IS* ischemic stroke, *TIA* transient ischemic attack, *SE* systemic embolism, *MI* myocardial infarction, *ICH* intracranial hemorrhage, *ECH* extracranial hemorrhage (either abnormal uterine bleed or other form of extracranial hemorrhage)

A decision-analytic Markov model with annual cycles and a lifetime horizon was developed in which VKAs and RVX were compared as treatments for the prevention of stroke in premenopausal women with AF. VKAs were provided in adjusted doses (target INR between 2.0 and 3.0) and RVX in a dose of 20 mg. The model included five different health states – “no history of stroke”, “previous stroke or TIA”, “previous stroke and minor disability”, “previous stroke and major disability”, and “death” – and nine clinical events: ischemic stroke, TIA, systemic embolism, myocardial infarction, intracranial hemorrhage, major and minor abnormal uterine bleeds (AUBs), and other major and minor extracranial hemorrhages. In each cycle, women from the hypothetical cohort remained in their health state or moved from one health state to another when a TIA, ischemic stroke, intracranial hemorrhage, or death occurred. See Fig. 1 for an overview of the model.

### Population

At baseline, the cohort of women in our analysis was 20 years of age and 0.5% had a history of stroke. This estimate was based on consultation of clinical experts. Menopause was assumed to set in at age 51 years. After this age, the occurrence of abnormal uterine bleeds is less common in women, although they do occur. In the model we conservatively assumed that after 51 years of age, women were no longer at risk for abnormal uterine bleeds. Based on the age distribution of premenopausal women in the Netherlands in 2015 and different studies on the prevalence of AF in age subgroups, [1, 2, 17, 18] we estimated that 10,000 premenopausal women had AF in The Netherlands in 2015, of which 10.5% were aged 20–29 years, 20% were 30–39, 49% were 40–49, and 20.5% were 50–51 years. We assumed that around 40% of these women were eligible for oral anticoagulation due to their co-morbidities [2]. See Additional file 1 (including Table S1) for more details.

This study focuses on a simulation model of a hypothetical cohort of premenopausal women with AF. Data on this cohort was based solely on publicly available data. This study was therefore not submitted to an institutional ethics committee. Approval from an ethics committee is required in The Netherlands only when scientific research subjects persons to at least one intervention or imposes on them a form of behavior, as stated in the Medical Research Involving Human Subjects Act.

### Probabilities

Clinical event rates were retrieved from different studies on the phase III trial on RVX [8, 19, 20] as well as a post approval study on RVX and VKAs for the prevention of venous thromboembolism in premenopausal women [8, 15]. The study population from the ROCKET-AF trial differed from the hypothetical cohort of premenopausal women in terms of gender, age, and history of stroke. This was important to consider as these variables influence the risk of different clinical events with VKAs as well as the relative risks with RVX. Adjustments were made for different clinical events on the basis of gender, age and history of stroke using different sources [8, 19–23]. The clinical event rates and risk adjustments that were used are listed in Additional file 1: Table S2.

### Utility

To assess whether RVX or VKAs form the preferred option, we needed to compare the benefit/risk profiles of both treatments. This is difficult because many different clinical events and health states are important to consider. However, these events and states can be transformed into a single utility measure – quality adjusted life years (QALYs) – and this measure was used to reflect the overall benefit/risk profiles. Quality of life was considered as a single index utility, on a scale from 0 (representing death) to 1 (representing perfect health). The decrement in utility caused by clinical events as well as the utility scores of health events were retrieved from different sources [24–26]. An overview of (dis)utilities is provided in Additional file 1: Table S3. QALYs were discounted at an annual rate of 1.5% [27].

### Costs

Health care costs were also used as input in the model. Costs and frequency of treatment/monitoring were collected from the websites of Dutch institutions [28, 29], and advise from clinical experts. Costs for clinical events and health states were obtained from health economic literature [30–33]. Price indices were used to convert costs to the 2015 price level [34]. Future costs were discounted to their present value by an annual rate of 4% [27]. An overview of the health care costs associated with treatment, monitoring, clinical events and health states can be found in the Additional file 1.

### Monte Carlo simulation

We performed a Monte Carlo simulation to obtain insight into how the uncertainty on the model parameters impact utility and cost-effectiveness. In the simulation, we ran the Markov model 10,000 times for a hypothetical cohort of 10,000 women aged 20 years, whereby – for every iteration – parameter values for clinical event rates, utilities and costs were randomly selected from their uncertainty distributions. The averages, and the 95% confidence intervals of these averages, were calculated over the 10,000 iterations, as well as the average increments of RVX compared to VKAs. We also calculated in how many iterations RVX performed better than VKAs with regard to clinical events and QALYs.

Cost-effectiveness of RVX was expressed as the “incremental cost-effectiveness ratio” (ICER) and the “net monetary benefit” (NMB). The ICER is a standard cost-effectiveness measure that expresses the healthcare costs associated with gaining one QALY. It is calculated here by dividing the incremental costs of RVX by its incremental effects. The NMB is the monetary value assigned to the total amount of QALYs that is associated with a treatment, subtracted by the costs of the treatment. The monetary value that is assigned to QALYs differs per context; the unofficial value of €50,000 per QALY in the Netherlands was used in our analysis [27]. The treatment with the highest NMB is considered cost-effective.

### Value of information analysis

Through a value of information (VOI) analysis, one can assess what the impact is of making more informed decisions because uncertainty on what is the best treatment option is reduced. For this purpose, we estimated the “expected value of perfect information” (EVPI). The EVPI is the expected value of eliminating all parameter uncertainty, here expressed as the maximum in QALYs that can be gained. The EVPI is estimated by calculating for each of the 10,000 iterations in the Monte Carlo simulation the difference between QALYs with the treatment that is chosen under uncertainty and QALYs with the treatment that would be chosen if “true” parameter values were known [35].

Not all women who initiate treatment before their menopause are 20 years old (the baseline age in the base case analysis). Therefore, the EVPI was calculated for different baseline ages before menopause, namely 20, 30, 40 and 50 years. To estimate the population EVPI, we multiplied these age-dependent EVPIs with the estimated number of women in each age group in The Netherlands.

If the VOI analysis would reveal that further research is warranted, we further investigated which variables mainly contribute to the decision uncertainty. A Tornado plot is useful for this purpose, which shows for the most important model parameters how their uncertainty influences the results in terms of incremental QALYs.

## Results

### Monte Carlo simulation

On average, RVX provides better protection against thromboembolic events and intracranial hemorrhages than VKAs do, but is associated with a higher risk of all forms of extracranial hemorrhages (Table 1). However, for many clinical events there is a large uncertainty on which treatment provides better protection. For example, the risk of ischemic stroke is found to be, on average, lower with RVX than VKAs – i.e. 0.44 less ischemic strokes per 10 patients over their lifetime. However, according to the 95% uncertainty range, this incremental effect may be even bigger (down to 3.18 less ischemic strokes) but RVX may also be harmful, leading up to 2.22 *more* ischemic strokes.

**Table 1:** Outcomes of the Monte Carlo simulation in which rivaroxaban (RVX) is compared to vitamin K antagonists (VKAs) for the prevention of stroke in a hypothetical cohort of premenopausal women with atrial fibrillation over their lifetime

	Rivaroxaban (RVX)		Vitamin K antagonists (VKAs)		Increment of RVX vs. VKAs		Chance RVX performs better
	Mean	95% CI^a^	Mean	95% CI^a^	Mean	95% CI^a^	
Benefit/Risk profile							
Clinical events, per 1000 subjects over the lifetime							
Ischemic stroke or TIA	567	408 to 759	611	428 to 832	−44	−318 to +222	61%
Systemic embolism	87	36 to 169	102	47 to 182	−15	−110 to +84	63%
Myocardial infarction	319	190 to 496	362	228 to 533	−43	−141 to +49	84%
Intracranial hemorrhage	136	74 to 226	210	146 to 290	−74	−140 to −8	98%
Extracranial hemorrhage (ECH)							
Major AUB	928	57 to 1990	429	21 to 894	499	−5.83 to +1690	24%
Major other ECH	1023	685 to 1458	832	639 to 1060	191	−65 to +536	9%
Minor AUB	3872	2194 to 5739	1868	1442 to 2314	2004	227 to +3929	1%
Minor other ECH	3763	2670 to 5137	3401	2716 to 4188	362	−513 to +1436	23%
QALYs, per subject	30.48	26.89 to 33.86	29.91	26.31 to 33.34	0.57	−0.80 to 2.15	78%
Cost-effectiveness (×€1000, per subject)							
Healthcare costs	63.7	45.2 to 91.4	47.5	32.5 to 66.7	16.3	−6.1 to 43.1	8%
Costs per QALY gained^b^	–	–	–	–	28.5	–	60%^c^
Net monetary benefit^c^	1460	1276 to 1637	1448	1265 to 1625	12	−75 to 109	60%

^a^The lower bound of the range equals the 2.5th percentile, and the upper bound equals the 97.5th percentile

^b^Otherwise defined as the incremental cost-effectiveness ratio (ICER)

^c^The net monetary benefit (NMB) is the monetary value assigned to the total amount of QALYs that is associated with a treatment, subtracted by the costs of the treatment. We assumed that one QALY was valued with €50,000. The treatment with the highest NMB is considered cost-effective

The last column shows that, in the 10,000 iterations of the Monte Carlo simulation, RVX prevents more ischemic strokes than VKAs (around 61% of the time). Notable from Table 1 is that there is little uncertainty on which treatment is associated with less intracranial hemorrhages (RVX in 98% of the iterations), and less major non-AUB extracranial hemorrhages and minor AUBs (VKAs in 91% and 99% of the iterations respectively).

**Fig. 2:**
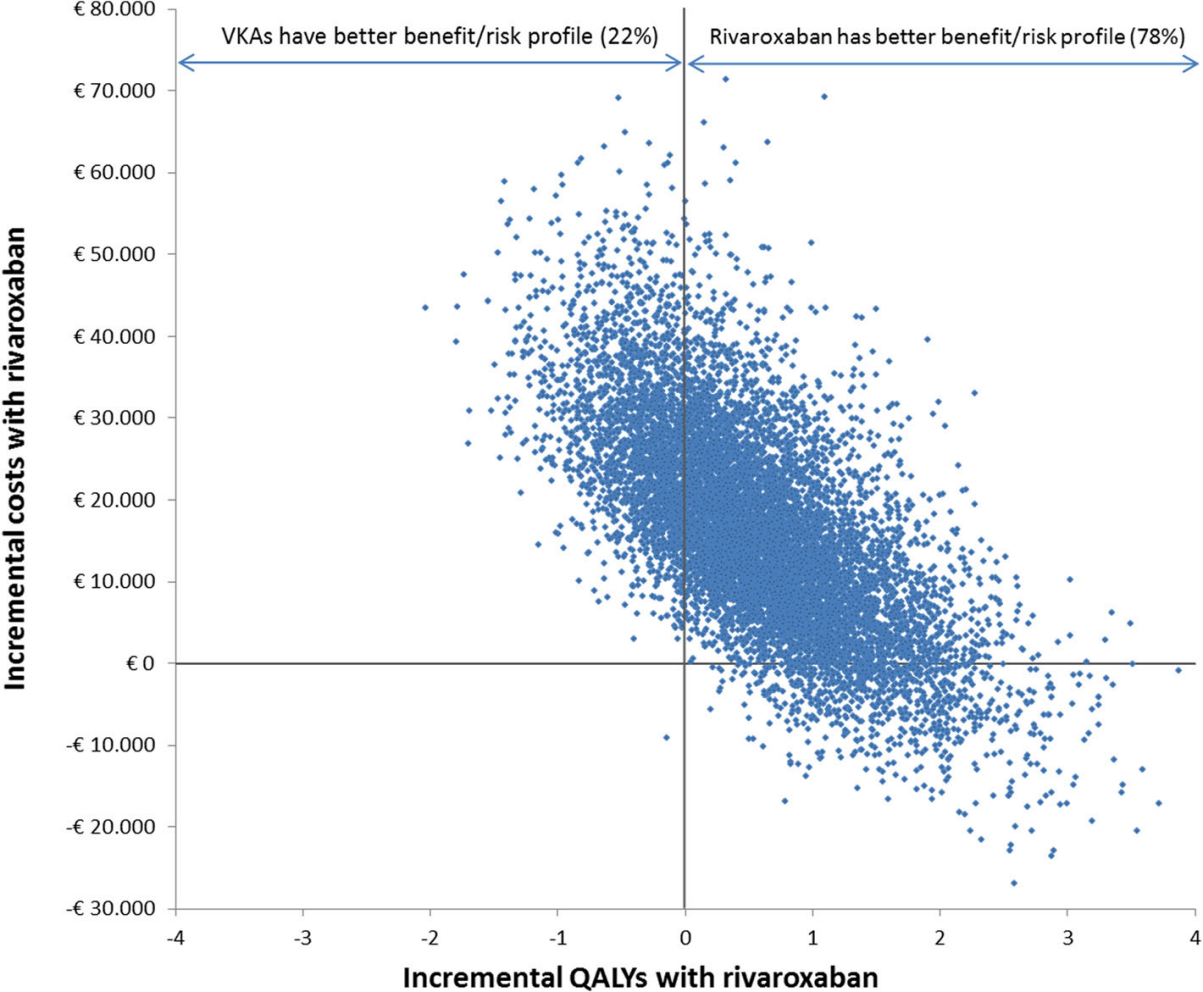
The probabilities that rivaroxaban leads to better or worse health (x-axis) than vitamin K antagonists (VKAs) in terms of Quality Adjusted Life Years (QALYs) and is more or less costly (y-axis). The figure is the result of the Monte Carlo simulation, in which the Markov model was iterated 10,000 times, whereby clinical event rates, utility scores and health care costs were randomly selected from their uncertainty distributions in each iteration

For each iteration in the simulation, the clinical events associated with each treatment were translated into a single health-related utility measure, namely QALYs. Treatment with RVX results, on average, in more QALYs than treatment with VKAs (30.48 vs. 29.91 per subject respectively). There is, however, a 22% chance that VKAs outperform RVX in this respect. This is also made visible in Fig. 2, which shows all the iterations from the Monte Carlo simulation, designating for each iteration the incremental QALYs (x-axis) and incremental costs (y-axis) with RVX as compared to VKAs.

RVX is associated with higher costs than VKAs in 92% of the iterations (Table 1). The mean increment of 0.57 QALYs with RVX comes at an average expense of €16,251. This implies that for every QALY gained with RVX, an additional expense of €28,506 is required. Assuming that within Dutch health care policy-makers are willing to pay €50,000 for each QALY gained, RVX has a 60% probability of being cost-effective.

### Value of information analysis

Table 2 shows for each 10-year age group before the age of 51 the estimated number of women in the Netherlands in 2015, as well the results from the VOI analysis. Visible from the table is that the VOI lowers with rising age. For each woman with a baseline age of 20, perfect information on effectiveness and safety yields 0.0849 QALY and has a net monetary benefit of €12,795 when compared with the current status of uncertainty. However, these values are 0.0076 and €1710 with a baseline age of 50. In the total group of premenopausal women in the Netherlands (2015), perfect information would improve women’s health with around 122 QALYs and would yield over 22 million Euros. The net monetary benefit represents the value of gaining QALYs and preventing healthcare costs because better decisions are being made.

**Table 2:** The value of reducing the decision uncertainty surrounding the choice between either rivaroxaban (RVX) or vitamin K antagonists (VKAs) in premenopausal women with atrial fibrillation in the Netherlands

	Per person			Total population		
	Estimated chance^a^	EVPI^b^		Estimated number	Population EVPI^b^	
		QALYs	NMB		QALYs	NMB
Baseline age^c^						
20 yrs	10.5%	0.0849	€12,795	420	35.7	€5,373,900
30 yrs	20%	0.0467	€8311	800	37.4	€6,648,800
40 yrs	49%	0.0219	€4430	1960	42.9	€8,682,800
50 yrs	20.5%	0.0076	€1710	820	6.2	€1,402,200
Total	100%	0.0305	€5527	4000	122.2	€22,107,700

^a^Represents the chance that the female patient with atrial fibrillation belongs to the respective age category

^b^Expected value of perfect information, which equals the outcomes (in terms of QALYs or NMB) when making treatment decisions under perfect information, subtracted with the outcomes when making decisions under current uncertainty. The EVPI consequently also equals the maximum value of information that can be gained with further research

^c^We assumed that the probability of having suffered a previous stroke increases with age: 0.5% in women aged 20; 1% in women aged 30; 2% in women aged 40; and 5% in women aged 50 years

**Fig. 3:**
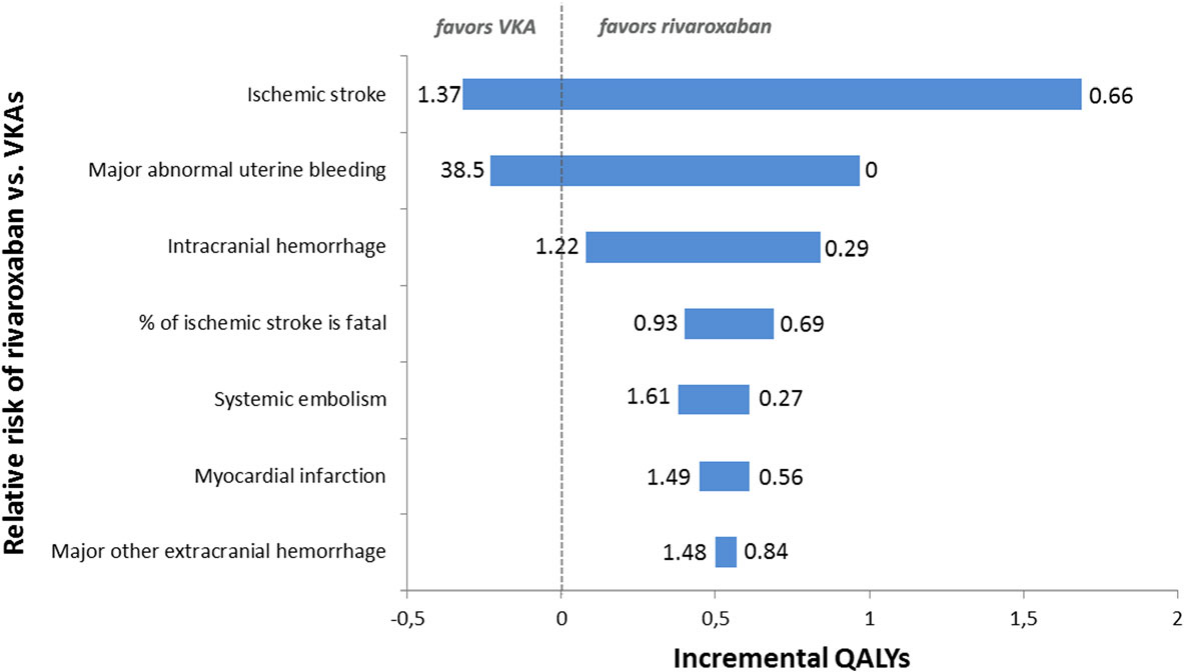
Tornado plot: Overview of the impact of the main health outcomes on decision uncertainty. The figure shows the impact of the current uncertainty on the relative risks (RRs) of rivaroxaban vs. Vitamin K Antagonists (VKAs) in premenopausal women with atrial fibrillation on Quality Adjusted Life Years (QALYs). For example, the RR of ischemic stroke with rivaroxaban vs. VKAs in premenopausal women currently ranges from 0.66 to 1.37. When the RR is 1.37, treatment with rivaroxaban leads to a loss of −0.32 QALYs. If the RR is 0.66, treatment with rivaroxaban leads to a gain of 1.69 QALYs

The tornado plot in Fig. 3 shows what the impact is of the uncertainty concerning the relative risks of RVX versus VKAs on incremental QALYs with RVX as compared to VKAs with regard to specific parameters. Uncertainty on the relative risk of ischemic stroke and major abnormal uterine bleeding has the most prominent impact on incremental QALYs with RVX. For example, under current uncertainty, the relative risk of ischemic stroke with RVX vs. VKAs in premenopausal women ranges from 0.66 to 1.37. If the relative risk would be 1.37, treatment with RVX will lead to a loss of −0.32 QALYs. If, on the other side of the spectrum, the relative risk equals 0.66, treatment with RVX will lead to a gain of 1.69 QALYs. Uncertainty on the relative risk of major non-AUB extracranial hemorrhages has the least impact of the parameters shown here; other parameters like the relative risk of minor hemorrhage, disutilities of clinical events, and utility scores of health states are not shown because the impact of their uncertainty is even smaller.

## Discussion

We set out to assess the decision uncertainty on whether RVX or VKAs should be prescribed in premenopausal women with AF. Although RVX is widely prescribed in this subgroup [11–13], first results suggested that the risk of AUB is higher with RVX than with VKAs whereas it may even be less effective in preventing ischemic strokes [19]. Using a model-based approach, we estimated that there is a 22% chance that the wrong decision is being made by prescribing RVX, implicating worse health outcomes than treatment with VKAs. This does not imply that there is sufficient reason to withhold RVX from premenopausal women – after all, RVX seems to have a 78% probability to have a better benefit/risk profile than VKAs. However, our study suggests that more research needs to be done for this subgroup because of the decision uncertainty.

Further research on the benefit/risk profile of RVX and VKAs in premenopausal women may add to decision-making by clinicians and policy-makers. We estimate that eliminating uncertainty will yield around 122 QALYs and has a value of over 22 million Euros in the Netherlands. The risks of ischemic stroke and major AUB provide the largest contribution to decision uncertainty, and should therefore be of primary concern in further research. As the number of premenopausal women with (paroxysmal) AF is relatively low and a large study population is required to gain enough statistical power to effectively investigate the risk of ischemic stroke, a large registry study is presumably the best option for further research, preferably on an international basis. Another reason to perform a registry study rather than a randomized controlled trial, is that there is currently no classical equipoise with regard to the right treatment. If other NOACs are prescribed in this population, these should also be included in the registry study.

The empirical evidence used in our model stems from various countries and clinical settings, therefore we believe that the results on the benefit/risk profile per individual are largely expandable to other countries and settings – although possible discrepancies in time in therapeutic with VKAs need to be considered. Our simulation model has several limitations. First, we could not include all currently available NOACs in our analysis. Although dabigatran, apixaban and edoxaban are also likely candidates for the prevention of stroke in premenopausal women with AF, we decided to restrict our analysis to RVX because this is the most prescribed NOAC. However, the bleeding risks that are specific for these women also hold for other NOACs. Therefore, if other NOACs are prescribed to premenopausal women, the events should also be registered in order to perform a comprehensive analysis in the future on which - if any - NOAC is preferred in these specific patients. Second, in general, results of modeling studies greatly depend on the choices made by the researchers, and results might therefore differ between various studies. For example, we assumed that treatment decisions were not associated with a decrement in quality of life. In contrast, several previous cost-effectiveness studies on NOACs did include such decrements [36–38]. We acknowledge that treatment with VKAs are potentially associated with more inconvenience, but do not consider this to have an impact on health-related quality of life. Also, self-measurement of INR may be very common under younger AF patients, and may be less costly than visits to thrombotic clinics. Because we did not include self-measurement in our analysis, the costs associated with treatment with VKAs may be overestimated, which means cost-effectiveness of RVX may also be overestimated.

Our study shows that the limited inclusion of premenopausal women in the phase III trial on RVX, and the omission of a subgroup analysis on them, has provoked decision uncertainty in clinical practice which may be potential harmful for them. This is related to the recurring fact that women are underrepresented in clinical trials, often leading to a false extrapolation of general results [39–41]. Of course, it is important to consider that specific subgroups may need to be excluded from phase III trials for ethical reasons, but the choice for patient exclusion is often not made explicit, raising questions on whether exclusion was justifiable [42, 43].

We propose a more frequent use of model-based studies to aid clinicians in decision making for optimal treatment when clinical study data are missing. Results from such an analyses may also be helpful for funding agencies or governmental bodies in prioritizing research [44, 45], also with the purpose of preventing wasteful studies [46].

## Conclusions

As AF often affects patients for the remainder of their life, it is important that the justified weighing of benefits and risks of long-term treatment are being made. We set out to assess the decision uncertainty surrounding a promising new treatment for AF in premenopausal women, a growing subgroup that has been overlooked in previous trials. Our study shows that although RVX seems promising, there is still uncertainty on whether RVX or VKAs should be prescribed in premenopausal women, mainly because of the uncertainty on the risk of AUBs and ischemic strokes. Further research on the use of NOACs in premenopausal women is warranted, and should preferably take the form of an internationally coordinated registry study. Estimating and reducing uncertainty on treatment decisions will benefit public health, and estimating the value of additional research may prevent additional wasteful research.

## Additional file

Additional file 1: Table S1. Overview of the number of women according to different age groups in the Netherlands in 2015, based on data from Statistics Netherlands, and used as input for the hypothetical cohort in the Markov model. **Table S2.** Relevant clinical event rates in patients with atrial fibrillation treated with rivaroxaban or vitamin K antagonists, as well as possible risk adjustments of these rates (for example based on gender and age), used as input in the Markov model. **Table S3.** Disutility scores associated with the clinical events associated with atrial fibrillation (such as stroke or myocardial infarction), as well as the utility scores associated with health states, used as input in the Markov model. **Table S4.** Costs associated with the treatment of patients with atrial fibrillation with rivaroxaban and vitamin K antagonists, clinical events, as well as health states, used as input in the Markov model. **Table S5.** Costs associated with the monitoring of treatment with rivaroxaban as well as with vitamin K antagonists, used as input in the Markov model. (DOCX 57 kb)

## Abbreviations

AF: Atrial fibrillation
AUB: Abnormal uterine bleed
ICER: Incremental cost-effectiveness ratio
NMB: Net monetary benefit
QALY: Quality adjusted life year
Rvx: Rivaroxaban
VKAs: Vitamin K antagonists
