# Supplementary material for: Uncertainty on the effectiveness and safety of rivaroxaban in premenopausal women with atrial fibrillation: empirical evidence needed
Source: BMC Cardiovasc Disord. 2017 Oct 13;17:260. doi: 10.1186/s12872-017-0692-1 (PMC5640919; doi:10.1186/s12872-017-0692-1)
Supplement: Additional file 1: Table S1. — Overview of the number of women according to different age groups in the Netherlands in 2015, based on data from Statistics Netherlands, and used as input for the hypothetical cohort in the Markov model. Table S2. Relevant clinical event rates in patients with atrial fibrillation treated with rivaroxaban or vitamin K antagonists, as well as possible risk adjustments of these rates (for example based on gender and age), used as input in the Markov model. Table S3. Disutility scores associated with the clinical events associated with atrial fibrillation (such as stroke or myocardial infarction), as well as the utility scores associated with health states, used as input in the Markov model. Table S4. Costs associated with the treatment of patients with atrial fibrillation with rivaroxaban and vitamin K antagonists, clinical events, as well as health states, used as input in the Markov model. Table S5. Costs associated with the monitoring of treatment with rivaroxaban as well as with vitamin K antagonists, used as input in the Markov model. (DOCX 57 kb) [file 12872_2017_692_MOESM1_ESM.docx]

**Additional file 1**

**Prevalence of atrial fibrillation in premenopausal women in The Netherlands**

In the Markov model, we set the youngest baseline age at 20 years. On average, menopause sets in at the age of 51 years. The table below shows the age distribution of premenopausal women in The Netherlands.

**Table S1. Number of women according to age group, the Netherlands, 2015 [1]**

| **Age** | **Total number** |
| --- | --- |
| 20-30 | 1,047,984 |
| 30-40 | 1,006,095 |
| 40-50 | 1,228,645 |
| 50-60^*^ | 1,207,855 |
| 20-60 | 4,490,579 |

* Of the women aged 50-60, 258,581 were 50 through 51 years of age.

There is no information on the prevalence of AF in premenopausal women in The Netherlands. A study performed in Sweden found that ±0.3% of women younger than 60 years were diagnosed in hospitals with AF [2]. In Sweden, ±22% of patients with symptomatic AF are not seen in the hospital, which implies that the actual prevalence of symptomatic AF in women younger than 60 years was ±0.4%. This would total 17,962 women in The Netherlands in 2015.

Based on the age distribution of women in 2015 in The Netherlands, and the assumptions that this age distribution equals that of Sweden and that the risk of developing AF doubles with every decade of age [3, 4], we estimate that the prevalence of AF is 0.1% in women aged 20-30 years, 0.2% in women aged 30-40 years, 0.4% in women aged 40-50 years, and 0.8% in women aged 50-60 years, equaling 1,050; 2,000; 4,900; and 2,050 in number respectively. On the basis of a Swedish study, we estimated that 40% of premenopausal women with AF had risk factors motivating oral anticoagulation [2].

**Clinical event rates**

**Table S2.** The relevant clinical event rates in patients with atrial fibrillation on rivaroxaban or vitamin K antagonists, as well as possible risk adjustments of these rates, used as input in the Markov model.

| **Variable** | **Base case** | **Range*** | **Reference** |
| --- | --- | --- | --- |
|  |  |  |  |
| **Ischemic stroke (IS)** |  |  |  |
| Vitamin K antagonists |  |  |  |
| Risk of stroke or systemic embolism (SSE) in women | 2.86% | 2.01–3.88% | [5] |
| Risk of IS as % of SSE | 82.20% | N/A | [5] |
| Risk adjustment: every 10 yrs age | 1.45 | 1.17–1.77 | [6] |
| Risk adjustment: no previous stroke | 0.72 | 0.44–1.08 | [7] |
| Risk adjustment: previous stroke | 1.28 | 0.90–1.70 | [7] |
| Ischemic stroke outcome |  |  |  |
| Death | 26.8% | 22.8–30.8% | [5, 8] |
| Major disability | 21.8% | 18.5–25.1% | [5, 8] |
| Minor disability | 30.9% | 26.3–35.5% | [5, 8] |
| No residual disability (=TIA) | 20.6% | N/A | [5, 8] |
| Rivaroxaban |  |  |  |
| RR of SSE vs. VKAs, all women | 0.88 | 0.60–1.22 | [5] |
| RR of SSE vs. VKAs, age < 65 yrs | 0.96 | 0.52–1.57 | [5] |
| RR of SSE vs. VKAs, age 65–74 | 0.89 | 0.59–1.27 | [5] |
| RR of SSE vs. VKAs, age ≥ 75 yrs | 0.82 | 0.55–1.16 | [5] |
| RR of IS vs. VKAs, no previous stroke | 0.88 | 0.52–1.36 | [7] |
| RR of IS vs. VKAs, previous stroke | 1.03 | 0.74–1.48 | [7] |
| Ischemic stroke outcome |  |  |  |
| Death | 21.7% | 18.4–25.0% | [5, 8] |
| Major disability | 20.3% | 17.3–23.3% | [5, 8] |
| Minor disability | 34.8% | 29.6–40.0% | [5, 8] |
| No residual disability (=TIA) | 23.2% | N/A | [5, 8] |
| **Systemic embolism (SE)** |  |  |  |
| Vitamin K antagonists |  |  |  |
| Risk of SE, no previous stroke | 0.16% | 0.03–0.44% | [7] |
| Risk of SE, previous stroke | 0.26% | 0.08–0.58% | [7] |
| Rivaroxaban |  |  |  |
| RR of SE vs. VKAs, no previous stroke | 0.91 | 0.14–2.61 | [7] |
| RR of SE vs. VKAs, previous stroke | 0.64 | 0.13–1.67 | [7] |
| Risk SE is fatal (for both VKAs and RVX) | 7% | N/A | [8] |
| **Myocardial infarction (MI)** |  |  |  |
| Vitamin K antagonists |  |  |  |
| Risk of MI | 1.12% | 0.78–1.52% | [7] |
| Risk adjustment: women vs. men | 0.89 | 0.57–1.01 | [5, 8] |
| Risk adjustment: every 10 yrs age | 1.43 | 1.18–1.78 | [7] |
| Risk adjustment: no previous stroke | 1.21 | 0.75–1.81 | [7] |
| Risk adjustment: previous stroke |  |  |  |
| Rivaroxaban |  |  |  |
| RR of MI vs. VKAs, no previous stroke | 0.77 | 0.44–1.21 | [7] |
| RR of MI vs. VKAs, previous stroke | 1.13 | 0.66–1.75 | [7] |
| Risk MI is fatal |  |  |  |
| Age 20–60 yrs | 4% | N/A | [9] |
| Age 60–70 yrs | 6% | N/A | [9] |
| Age 70–80 yrs | 7% | N/A | [9] |
| Age 80+ yrs | 18% | N/A | [9] |
| **All non-AUB major hemorrhages** |  |  |  |
| Vitamin K antagonists |  |  |  |
| Risk adjustment: women vs. all | 0.90 | 0.64–1.20 | [5] |
| Risk adjustment: every 10 yrs age | 1.61 | 1.40–1.85 | [6] |
| Rivaroxaban |  |  |  |
| Risk adjustment: RR of women vs. all | 0.96 | 0.69–1.29 | [5] |
| RR of MH vs. VKAs, age < 65 yrs | 1.02 | 0.59–1.59 | [5] |
| RR of MH vs. VKAs, age 65–74 | 0.91 | 0.65–1.23 | [5] |
| RR of MH vs. VKAs, age ≥ 75 yrs | 1.15 | 0.89–1.45 | [5] |
| **Intracranial hemorrhage (ICH)** |  |  |  |
| Vitamin K antagonists |  |  |  |
| Risk of ICH, no previous stroke | 0.68% | 0.34–1.17% | [7] |
| Risk of ICH, previous stroke | 0.80% | 0.43–1.30% | [7] |
| Rivaroxaban |  |  |  |
| RR of ICH vs. VKAs, no previous stroke | 0.57 | 0.21–1.15 | [7] |
| RR of ICH vs. VKAs, previous stroke | 0.74 | 0.36–1.29 | [7] |
| ICH outcome (VKAs and rivaroxaban) |  |  |  |
| Death | 49% | N/A | [8] |
| Major disability | 43% | N/A | [8] |
| Minor disability | 8% | N/A | [8] |
| **Non-AUB major extracranial hemorrhage (NMECH)** |  |  |  |
| Vitamin K antagonists |  |  |  |
| Risk of NMECH, no previous stroke | 3.01% | 2.21–3.95% | [7] |
| Risk of NMECH, previous stroke | 2.42% | 1.74–3.23% | [7] |
| Rivaroxaban |  |  |  |
| RR of NMECH vs. VKAs, no previous stroke | 1.23 | 0.94–1.58 | [7] |
| RR of NMECH vs. VKAs, previous stroke | 1.05 | 0.76–1.39 | [7] |
| Risk NMECH is fatal (VKAs and rivaroxaban) | 7% | N/A | [10] |
| **Non-AUB non-major ECH (NNECH)** |  |  |  |
| Vitamin K antagonists |  |  |  |
| Risk of NNECH, no previous stroke | 11.78% | 10.20–13.47% | [7] |
| Risk of NNECH, previous stroke | 10.98% | 9.51–12.56% | [7] |
| Rivaroxaban |  |  |  |
| RR of NNECH vs. VKAs, no previous stroke | 1.10 | 0.96–1.25 | [7] |
| RR of NNECH vs. VKAs, previous stroke | 0.99 | 0.86–1.13 | [7] |
| **Major abnormal uterine bleed (MAUB)** |  |  |  |
| Vitamin K antagonists | 0.16% | 0–6.3% | [11, 12] |
| Rivaroxaban | 1.54% | 0–7.7% | [11, 12] |
| Risk MAUB is fatal (VKAs and rivaroxaban) | 7% | N/A | [10] |
| **Non-major abnormal uterine bleed (NAUB)** |  |  |  |
| Vitamin K antagonists | 6.34% | 3.30–10.2% | [11, 12] |
| Rivaroxaban | 12.55% | 6.93–19.61% | [11, 12] |
|  |  |  |  |

* Ranges represent 99.7% confidence intervals of the beta distributions.

In clinical practice, the success of VKA treatment is greatly dependent on the time patients spend “in therapeutic range.” Patients are considered in therapeutic range when their INR levels are between 2.0 and 3.0 or 3.5. The risk of thromboembolic and bleeding events are higher when outside this range. In the ROCKET-AF trial time in therapeutic range (TTR) was ±55%, and it was reported that the TTR is higher in The Netherlands, perhaps even close to 80% [10, 13, 14]. However, Dutch patients are considered within therapeutic range when their INR is between 2.0 and 3.5, while this range was 2.0–3.0 in the ROCKET-AF trial. It was estimated that, with a narrower definition (2.5–3.5), the TTR in The Netherlands was similar to that of the ROCKET-AF trial (±59%) [13]. Based on a subgroup analysis of the ROCKET-AF trial [15], it was considered likely that such a difference in TTR will have only have a minor impact on clinical event rates, if any. The clinical event rates of VKAs were therefore not adjusted for TTR.

Rates of major and minor AUB were taken from Martinelli et al., 2015 [15]. In this study, rates were reported separately for women receiving either hormonal therapy for birth control, or no hormonal therapy. We assumed that 32% of premenopausal women in the Netherlands used hormonal therapy [24]. We used the annual risks of major and minor AUB from this trial starting from the second month after randomization, because there was a large spike in bleeding events in the first month after randomization.

**Utility**

**Table S3**. Disutility scores associated with the clinical events associated with atrial fibrillation, as well as the utility scores associated with health states.

| **Variable** | **Value** | | **Time**  **frame** | **Reference** |
| --- | --- | --- | --- | --- |
|  | Base case | (Range) |  |  |
|  |  |  |  |  |
| **Disutilities scores associated with clinical events** | | | | |
| Transient ischemic attack (TIA) | –0.1032 | –0.0881 to 0.1189 | 1 month | [16] |
| Stroke/intracranial hemorrhage | –0.1385 | –0.1184 to 0.1601 | 6 months | [16] |
| Systemic embolism | –0.1199 | –0.1022 to 0.1388 | 6 months | [16] |
| Myocardial infarction | –0.1247 | –0.1065 to 0.1436 | 6 months | [16] |
| Major extracranial hemorrhage | –0.1814 | –0.1548 to 0.2090 | 1 month | [16] |
| Minor hemorrhage | –0.1814 | –0.1548 to 0.2090 | 2 days | [16, 17] |
|  |  |  |  |  |
| **Utility scores associated with health states and treatments** | | | | |
| No previous stroke or TIA | 0.81 | 0.66 to 0.97 | 1 year | [16] |
| Previous stroke/TIA, minor disability | 0.68 | 0.52 to 0.81 | 1 year | [18] |
| Previous stroke/TIA, major disability | 0.52 | 0.30 to 0.68 | 1 year | [18] |
|  |  |  |  |  |

**Health care costs**

**Table S4.** Costs associated with treatment, clinical events and health states in the Markov model.

| **Variable** | **Base case** | **Range** | **Reference** |
| --- | --- | --- | --- |
|  |  |  |  |
| **Drug costs (per unit)** |  |  |  |
| VKAs (Acenocoumarol 1 mg) | € 0.02 | NA | [19] |
| Rivaroxaban | € 2.29 | NA | [19] |
| **Drug intake frequency (annual)** |  |  |  |
| VKAs | 1,095 | 730 – 1,460^*^ | [20] |
| Rivaroxaban | 365 | NA |  |
| **Costs associated with clinical events** |  |  |  |
| Ischemic stroke |  |  |  |
| <60 yrs | € 22,655 | ±50%^†^ | [21] |
| 60-70 yrs | € 25,325 | “ | [21] |
| 70-80 yrs | € 28,976 | “ | [21] |
| 80-90 yrs | € 30,299 | “ | [21] |
| 90+ yrs | € 30,640 | “ | [21] |
| Systemic embolism | € 1,075 | “ | [10] |
| Myocardial infarction | € 19,781 | “ | [22] |
| Intracranial hemorrhage | € 29,267 | “ | [10, 21] |
| Extracranial hemorrhage (AUB or non-AUB) | € 16,243 | “ | [23] |
| Minor hemorrhage (AUB or non-AUB) | € 37 | “ | [24] |
| **Costs associated with health states** |  |  |  |
| Prior stroke, disability |  |  |  |
| <60 yrs | € 5,031 | “ | [21] |
| 60-70 yrs | € 5,679 | “ | [21] |
| 70-80 yrs | € 6,571 | “ | [21] |
| 80-90 yrs | € 6,935 | “ | [21] |
| 90+ yrs | € 7,054 | “ | [21] |
|  |  |  |  |

* Normal distribution, the assumption is that patients on VKAs take 3x Acenocoumarol 1 mg daily on average, ranging from 2x per day to 4x per day.

† Gamma distribution, percentage of range is a broad assumption.

**Table S5**. Costs input of treatment monitoring

| **Variable** | **General practitioner** | **General hospital** | **Academic**  **hospital** | **Thrombotic clinic** | **Ref** |
| --- | --- | --- | --- | --- | --- |
|  |  |  |  |  |  |
| **Costs of visit (per unit)** | €34 (NA) | €81 (NA) | €165 (NA) | €10.78 (€5–€20)* | [25, 26] |
|  |  |  |  |  |  |
| **Frequency of visits (annual)†** |  |  |  |  |  |
| Monitoring of treatment, 1^st^ yr | - | 1.5 (0.75–2.25) | 0.5 (0.25–1.25) | - | ‡ |
| Regular monitoring with AF | 0.3 (0–0.6) | 0.15 (0–0.3) | 0.05 (0–0.1) | - | ‡ |
| VKAs: INR monitoring, 1^st^ yr | - | - | - | 24 (12–36) | [26] |
| VKAs: regular monitoring | - | - | - | 21 (12–30) | [26] |
|  |  |  |  |  |  |

* Gamma distribution.

† Normal distribution, lower range ≥ 0.

‡ Assumption.

**References**

1. Statistics Netherlands. CBS Statline: [http://statline.cbs.nl/statweb. Accessed 16 Sep 2016](http://statline.cbs.nl/statweb.%20Accessed%2016%20Sep%202016).
2. Ball J, Carrington MJ, McMurray JJ, Stewart S. Atrial fibrillation: profile and burden of an evolving epidemic in the 21^st^ century. Int J Cardiol. 2013;167:1807‒24
3. Renoux C, Patenaude V, Suissa S. Incidence, mortality, and sex differences of non-valvular atrial fibrillation: a population-based study. J Am Heart Assoc. 2014;3:e001402.
4. Friberg L, Bergfeldt L. Atrial fibrillation prevalence revisited. J Intern Med. 2013;275:461‒468.
5. Johnson & Johnson Pharmaceutical Research & Development. Advisory committee briefing document. Rivaroxaban for the prevention of stroke and non-central nervous system (CNS) systemic embolism in patients with atrial fibrillation. 2011. http://www.fda.gov/downloads/AdvisoryCommittees/CommitteesMeetingMaterials/Drugs/CardiovascularandRenalDrugsAdvisoryCommittee/UCM270797.pdf. Accessed 20 Dec 2016.
6. Van Walraven C, Hart RG, Connolly S, Austin PC, Mant J, Hobbs FD, et al. Effect of age on stroke prevention therapy in patients with atrial fibrillation: the atrial fibrillation investigators. Stroke. 2009;40:1410‒6.
7. Hankey GJ, Patel MR, Stevens SR, Becker RC, Breithardt G, Carolei A, et al. Rivaroxaban compared with warfarin in patients with atrial fibrillation and previous stroke or transient ischaemic attack: a subgroup analysis of ROCKET AF. Lancet Neurol. 2012;11:315‒22
8. Fang MC, Go AS, Chang Y, Hylek EM, Henault LE, Jensvold NG, et al. Death and disability from warfarin-associated intracranial and extracranial hemorrhages. Am J Med. 2007;120:700‒5.
9. Leurent G, Garlantézec R, Auffret V, Hacot JP, Coudert I, Filippi E, et al. Gender differences in presentation, management and inhospital outcome in patients with ST-segment elevation myocardial infarction: data from 5000 patients included in the ORBI prospective French regional registry. Arch Cardiovasc Dis. 2014;107:291‒8.
10. Verhoef TI, Redekop WK, Hasrat F, de Boer A, Maitland-van der Zee AH. Cost effectiveness of new oral anticoagulants for stroke prevention in patients with atrial fibrillation in two different European healthcare settings. Am J Cardiovasc Drugs. 2014;14:451‒62.
11. Picavet C. Zwangerschap en anticonceptie in Nederland [Pregnancy and contraception in the Netherlands]. Tijdschrift voor Seksuologie. 2012;36:121‒8.
12. Martinelli I, Lensing AW, Middeldorp S, Levi M, Beyer-Westendorf J, van Bellen B, et al. Recurrent venous thromboembolism and abnormal uterine bleeding with anticoagulant and hormone therapy use. Blood. 2016;127:1417‒25.
13. Bezemer ID, Roemer WH, Penning-van Beest FJ, van Eekelen E, Kramer MH. INR control calculation: comparison of Dutch and international methods. Neth J Med. 2013;71:194‒8.
14. Federation of Dutch Anticoagulant clinics. Samenvatting medische jaarverslagen 2014. http://www.fnt.nl/media/docs/FNT_Samenvatting_Medisch_JV_2014.pdf. Accessed 16 Sep 2016.
15. Piccini JP, Hellkamp AS, Lokhnygina Y, Patel MR, Harrell FE, Singer DE, et al. Relationship between time in therapeutic range and comparative treatment effect of rivaroxaban and warfarin: results from the ROCKET AF trial. J Am Heart Assoc. 2014;22:e000521.
16. Sullivan PW, Arant TW, Ellis SL, Ulrich H. The cost effectiveness of anticoagulation management services for patients with atrial fibrillation and at high risk of stroke in the US. Pharmacoeconomics. 2006;24:1021‒33.
17. O’Brien CL, Gage BF. Costs and effectiveness of ximelagatran for stroke prophylaxis in chronic atrial fibrillation. JAMA. 2005;293:699‒706.
18. Tengs TO, Lin TH. A meta-analysis of quality-of-life estimates for stroke. Pharmacoeconomics. 2003;21:191‒200.
19. Dutch drug costs. Available from: http://www.medicijnkosten.nl.
20. The National Health Care Institute [Zorginstituut Nederland]. Pharmacotherapeutic compass. Available from: http://www.farmacotherapeutischkompas.nl.
21. Struijs JN, van Genugten ML, Evers SM, Ament AJ, Baan CA, van den Bos GA. Future costs of stroke in The Netherlands: the impact of stroke services. Int J Technol Assess Health Care 2006;22:518–524.
22. Greving JP, Visseren FLJ, De Wit GA, Algra A. Statin treatment for primary prevention of vascular disease: whom to treat? Cost-effectiveness analysis. BMJ 2011;342:d1672.
23. De Leest HT, Van Dieten HE, Van Tulder MW, Lems WF, Dijkman BA, Boers M. Costs of treating bleeding and perforated peptic ulcers in The Netherlands. J Rheumatol 2004;31:788‒791.
24. Mensch A, Stock S, Stollenwerk B, Müller D. Cost effectiveness of rivaroxaban for stroke prevention in German patients with atrial fibrillation. Pharmacoeconomics 2015;33:271‒283.
25. The National Health Care Institute [Zorginstituut Nederland]. Kostenhandleiding: Methodologie van kostenonderzoek en referentieprijzen voor economische evaluaties in de gezondheidszorg. https://www.zorginstituutnederland.nl/binaries/content/documents/zinl-www/documenten/publicaties/

overige-publicaties/1602-richtlijn-voor-het-uitvoeren-van-economische-evaluaties-in-de-gezondheidszorg-bijlagen/1602-richtlijn-voor-het-uitvoeren-van-economische-evaluaties-in-de-gezondheidszorg-bijlagen/ Richtlijn+voor+het+uitvoeren+van+economische+evaluaties+in+de+gezondheidszorg+(verdiepings-modules).pdf. Accessed 16 Sep 2016.

1. Federation of Dutch Anticoagulant clinics. Samenvatting medische jaarverslagen 2014. http://www.fnt.nl/media/docs/FNT_Samenvatting_Medisch_JV_2014.pdf. Accessed 16 Sep 2016.
